# Improving interpretation of publically reported statistics on health and healthcare: the Figure Interpretation Assessment Tool (FIAT-Health)

**DOI:** 10.1186/s12961-018-0279-z

**Published:** 2018-03-07

**Authors:** Reinie G. Gerrits, Dionne S. Kringos, Michael J. van den Berg, Niek S. Klazinga

**Affiliations:** 1 Department of Public Health, Academic Medical Center, University of Amsterdam, Amsterdam, The Netherlands; 2 National Institute for Public Health and the Environment (RIVM), Bilthoven, The Netherlands

**Keywords:** Evidence-informed decision-making, Science communication, Scientific reporting

## Abstract

**Background:**

Policy-makers, managers, scientists, patients and the general public are confronted daily with figures on health and healthcare through public reporting in newspapers, webpages and press releases. However, information on the key characteristics of these figures necessary for their correct interpretation is often not adequately communicated, which can lead to misinterpretation and misinformed decision-making. The objective of this research was to map the key characteristics relevant to the interpretation of figures on health and healthcare, and to develop a Figure Interpretation Assessment Tool-Health (FIAT-Health) through which figures on health and healthcare can be systematically assessed, allowing for a better interpretation of these figures.

**Methods:**

The abovementioned key characteristics of figures on health and healthcare were identified through systematic expert consultations in the Netherlands on four topic categories of figures, namely morbidity, healthcare expenditure, healthcare outcomes and lifestyle. The identified characteristics were used as a frame for the development of the FIAT-Health. Development of the tool and its content was supported and validated through regular review by a sounding board of potential users.

**Results:**

Identified characteristics relevant for the interpretation of figures in the four categories relate to the figures’ origin, credibility, expression, subject matter, population and geographical focus, time period, and underlying data collection methods. The characteristics were translated into a set of 13 dichotomous and 4-point Likert scale questions constituting the FIAT-Health, and two final assessment statements. Users of the FIAT-Health were provided with a summary overview of their answers to support a final assessment of the correctness of a figure and the appropriateness of its reporting.

**Conclusions:**

FIAT-Health can support policy-makers, managers, scientists, patients and the general public to systematically assess the quality of publicly reported figures on health and healthcare. It also has the potential to support the producers of health and healthcare data in clearly communicating their data to different audiences. Future research should focus on the further validation of the tool in practice.

## Background

Every day, numerous figures related to health and healthcare are reported in all kinds of sources. Policy-makers, managers, scientists, patients and the general public use these figures to guide their thinking on topics of health and healthcare [1–4]. Based on these figures, inferences are made on the severity, magnitude or impact of a health issue in society [5], influencing the decision-making process of patients [6, 7] and public opinion, which is central to priority-setting in health policy [8] and science [9].

Ideally, people base their decisions on the best available evidence, retrieving the figures which support their thinking directly from the source in which the figures are initially published; however, this is often not the case [10–12]. When looking for information on health and healthcare, people will obtain information from secondary sources and organisations they deem reliable [13], such as sources found through internet searches, newspapers, information leaflets of consumer organisations, television programmes and scientific information provided by research institutes [14–17].

During the construction process of figures on health and healthcare, choices are made on definitions of what is counted and measured, which inclusion and exclusion criteria are used, and which methodology is applied; moreover, interests of the involved parties may influence the results [18]. Figures on health and healthcare are often reproduced and cited in reports, summaries, fact sheets, press releases and news messages. Through this process, mistakes and misunderstandings may easily occur, or figures may be deliberately manipulated [19–21]. Inadequate communication of the construction of these figures may result in a misreporting of estimates such as prevalence, disease severity and outcomes of research [3, 22, 23], eventually leading to wrongful interpretation of figures by readers of such publications [24]. Furthermore, multiple sources of information may report contradicting figures on the same topic [25]. Contradictory or unclear reporting may cause uncertainty regarding a health(care) topic [26, 27], creating a barrier [28, 29] or leading to avoidance of decision-making, or even lead to misinformed decisions [30, 31]. In addition, figures on similar topics leave room for political and anecdotal use, such as applying the figure that fits best with the agenda of its users [31, 32] (e.g. politicians [33], patient advocacy of patient organisations [34], or media generating attention by publishing negative figures [35]).

Increasingly, attention is being paid to the translation of evidence into policy and practice [36], as illustrated, for instance, by the development of the AGREE instrument, which assesses the quality of the process and reporting of clinical practice guidelines [37], the AIRE instrument, which assesses the methodological quality of healthcare indicators and the connected reports [38], and the GATHER statement, which assesses the reporting practice of global health estimates [39]. Such instruments, which seem to be actively used in practice, are aimed to give an in-depth assessment of the quality of research outcomes and the detailed reporting of health estimates.

Methods aimed at the in-depth assessment of figures reported in scientific or extensive research publications are widely available. However, there is a lack of methods through which a practical assessment can be made of publically reported statistics. Journalists, policy-makers or interested citizens may question whether they can trust certain figures, but often do not have the time or inclination to dive into the world of statistics and research methodology. A need exists for an easy to use tool that supports users to gain insight into the key characteristics that contribute to the interpretation of a figure on health or healthcare [40–42]. The objectives of this study are to (1) map the key characteristics relevant to the interpretation of figures on health and healthcare, and (2) to develop a Figure Interpretation Assessment Tool (FIAT-Health) enabling systematic assessment of publically reported figures on health and healthcare to improve the proper use of these figures by policy-makers, managers, scientists, patients and the general public.

## Methods

The design of the FIAT-Health was guided by a qualitative approach, relying on data derived from various forms of expert consultation in the Netherlands.

### Four topic categories of figures on health and healthcare

A broad range of data is available on health and healthcare, resulting in numerous figures of varying origin. This study focusses on four categories of figures, selected by the research group (the authors) because they differ strongly in their nature and type of data required, but are all commonly used in health policy, monitoring and healthcare planning. Moreover, these types of figures are found daily in the media by the general public. The wide range of figures covered by these categories is likely to generate insight with regards to the most essential characteristics of figures that are important for their interpretation, and are also generalisable to other figures on health and healthcare.

#### Morbidity figures

Morbidity figures are key in determining the incidence and prevalence of diseases in a population, forming the first ideal step to estimate the need for healthcare [43]. Figures on morbidity are typically collected through various sources, such as clinical registry data and health surveys.

#### Healthcare expenditure figures

Planning of healthcare services relies on the affordability of care, which is estimated through figures on healthcare expenditure [44, 45]. Depending on the type of system, expenditure data may be based on tax data, insurance claims, providers’ balance sheets, etc. Cost may, for example, be presented in relation to the GDP, per person, or in relation to certain diseases or types of care. In this category, figures are often the result of modelling.

#### Healthcare outcome figures

The quality of healthcare services is often perceived through figures on healthcare outcomes. Through these figures, the need for healthcare quality improvement and action on health is determined [46, 47]. This includes clinical outcomes, such as readmissions or complications and patient-reported outcomes. Data on healthcare outcomes is mostly derived through various clinical registries, administrative databases and patient surveys.

#### Lifestyle figures

Lifestyle is an important determinant of health, which is needed to estimate changes in the health of a population [48]. Often used examples are figures regarding physical activity, diet, and the use of alcohol, tobacco and drugs. Quantitative information on lifestyle is mostly collected using surveys and increasingly by wearable devices [49]. For macro-level figures, turnover of certain industries (e.g. tobacco) can also be used.

### Data collection

**Fig. 1:**
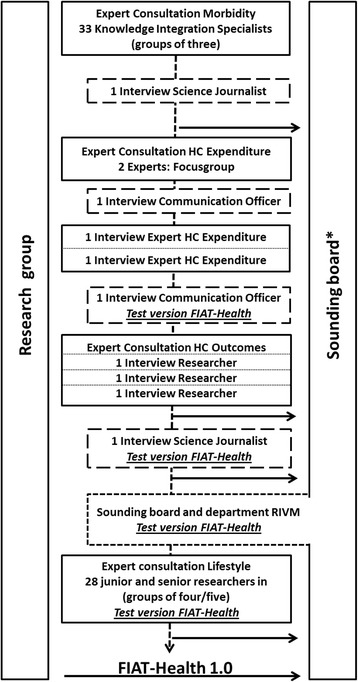
Data collection process

The research took place in the Netherlands, involving experts from four academic institutions and one national public health institute. Through purposeful sampling [50], researchers and *knowledge integration specialists* who specialise in communicating research findings to policy-makers and the public, were selected, based on their extended expertise in the respective topic categories. Furthermore, science journalists and communication officers were consulted for their experience with reporting figures in the media. Expert consultations were carried out in various formats, constructed according to the emerging knowledge need and participation of experts, as outlined below. The data collection process is described in Fig. 1. Each consultation meeting lasted between 60 and 90 minutes. By taking notes during the meetings and adding written comments made by the participants, the first author compiled a report for each session.

#### Stage 1

The first stage concerned the gathering of a broad list of characteristics on the practice of reporting figures. Data on the characteristics was obtained through the expert consultation for the morbidity category involving 33 specialists in the integration of knowledge on health and healthcare from the National Institute for Public Health and the Environment (RIVM). Fourteen groups of three experts each were asked to review the figures on a disease on four national websites from the RIVM [51–54]. To make groups of three, nine experts participated in two different groups. Experts were asked to note the type of prevalence or incidence, the population, year, sex, age, source of the figure and the source of the data on which the figure was based. They were asked to reflect on the clarity of the figures, give their opinion on the current publication practices, identify inconsistencies that they found between the collected figures across the four websites, and identify what information was needed to improve interpretation of the figures. Each group gathered face-to-face and gave a joint written response in a pre-structured Excel file. Their notes were compiled by the first author in a separate Excel file and summarised.

#### Stage 2

As in the first stage, a broad list of characteristics was derived, the second stage concerned the addition of characteristics particular to the category healthcare expenditure. A group process including a discussion was considered to provide deeper insight into these characteristics. Four senior health economists from two health economics research departments and the RIVM were invited. All four experts accepted the invitation, although only two were finally able to attend the group session. During the session, the participants were asked to note their thoughts on the interpretation of presented figures on healthcare expenditure supported by an example of a report; consequently, the notes were structured during the session and recorded by the two present researchers. Separate appointments were made with the participants who did not attend the first session. During these individual consultations, the findings of the first meeting were discussed in-depth, complemented and validated.

#### Stage 3

Based on the previous stages, an extended characteristics list was developed. At this stage, more in-depth questioning on the construction of figures was sought. These questions were asked during the expert consultation for the healthcare outcomes category. The consultation consisted of two in-person interviews and one phone interview with three senior researchers from two academic institutions and one private company. Open questions with a semi-structured format were used to gain insight into the considerations made during both the construction and use of figures on healthcare outcomes.

#### Stage 4

At the fourth stage, an early version of the tool was developed, and gaps in the list of the items needed to be identified and phrasing needed to be clarified. Making use of the available expertise at the research institute, the expert consultation for the lifestyle category was addressed through a meeting with 28 junior and senior researchers of a Public Health research department. Participants received a publication on a lifestyle figure. Three groups assessed the figure through an early version of the FIAT-Health, while three other groups assessed the figure without the structured support of the draft FIAT-Health. Their findings and experiences with assessing the figure were compared and discussed in a plenary session.

Furthermore, two science journalists writing for two online journalist platforms, and two communication officers from the RIVM were individually consulted on their experience with the communication of figures on health and healthcare towards the public. Both journalists had specific knowledge on the communication of figures on health and healthcare. The consultations were aimed at the use of figures in the media, and they shared their experience on how they make figures on health and healthcare understandable to the public and policymakers.

### Data analysis

Based on the expert consultations [55], insight was gained through an inductive approach in the key characteristics of each of the four topic categories of figures on health and healthcare that are relevant to their interpretation. First, to gain an overview of characteristics relevant to the interpretation of figures, characteristics related to the use of figures and those related to the construction of figures were coded in the session reports by the first author, resulting in an overview of characteristics for each of the four categories, and were successively compiled into a single list. Finally, characteristics in this list were synthesised resulting in the key characteristics that are essential to the interpretation of most figures on health and healthcare.

The format of the expert consultations differed among the categories in terms of approach and number of participants. Instead of first collecting all data in a single stage, an iterative process was applied during which multiple draft versions of the FIAT-Health were developed over time based on the emerging characteristics and shared with the involved experts. Hence, in the fourth stage, during the final interview with a communication officer and the final interview with a science journalist, a preliminary draft to which participants could respond was presented, which allowed the testing of insights gained from prior sessions in subsequent consultations.

### Development of the FIAT-Health

The key characteristics were structured in eight overarching themes that formed the basis of tool development, guiding the development of the content, form and outcome of the FIAT-Health. The themes comprised the structure on which the main components of the tool were based. Starting from this structure, the components suitable to address the themes were created.

The development of the tool was guided by constant dialogue amongst the research group, consisting of the authors of this paper. Furthermore, to gain insight into the expectations and needs of potential users of the tool, nine project leaders of the RIVM, who are highly experienced in the publication of figures on health and healthcare, were gathered in a sounding board. This sounding board supported the development of the tool by advising on the form and regularly reviewing its content. The panel gathered five times, during which draft versions of the tool were reviewed in detail. Feedback resulting from these meetings was used to adapt and refine the FIAT-Health. During one additional meeting, an early version of the instrument was tested by the sounding board and several knowledge integration specialists at the RIVM. In small groups they assessed an example of a published figure and reflected on the results.

Face validity was established through examination by the research group of the relevance, reasonability, unambiguousness and clarity of the content [55]. Content validity was assured through the review of the tool on both content as well as form by the sounding board, thereby assuring a balanced inclusion of the aspects relevant for the interpretation of figures on health and healthcare [55].

## Results

Characteristics relevant to the interpretation of figures on health and healthcare were mapped for the four categories. Interpretation was considered to consist of characteristics relevant to the construction and use of a figure. The construction of a figure relates to the methodological considerations that impact the quality of a figure, and characteristics relevant to the use of a figure relates to the information which is needed to apply the figure in practice. As the objective was to identify the characteristics relevant to the interpretation of figures, only the characteristics related to the construction of the figure that could be understood by those without specialised knowledge were included. Furthermore, characteristics that were relevant to the use of a figure on health and healthcare and which related to the information the user of a figure would need to assess the usability of a figure in a certain context were also included. The resulting key characteristics that were relevant for the interpretation of each of the four categories of figures were grouped into eight themes.

### Themes

#### Origin of the figure

This refers to the primary publication in which the figure was published for the first time. Any (secondary) publication that cites or refers to the figure should properly refer to this primary publication. The primary publication needs to be accessible to confirm the construction of the figure.

#### Credibility of the figure

Credibility is, to a large extent, a subjective judgement of the reader or user of a figure. This judgement is based on the expected expertise of the author and on possible conflicts of interest. Regarding expertise, a peer-reviewed scientific article from a well-known research group leads to higher expectations of credibility than an unknown blogger. Any financial or political interests may bias results or may appear to do so.

#### Expression of the figure

Two categories are distinguished, namely singular figures, such as an average or an absolute number, and composite figures such as a percentage or a fraction. The characterisation of the figure will impact the way the figure is perceived by its reader. When a figure is communicated in a singular form, the context of that figure might be lost to its user, while a composite form could obscure the actual figure. Either way, the form will impact the way the reader will value a figure.

#### Subject to which the figure applies

The definition of the subject is a decision made by the authors of the publication. Often, several definitions of the seemingly same subject are available. A more broad or narrow definition can impact figures considerably. Moreover, a misconception of the definition of the subject will result in a wrongful interpretation.

#### Population to which the figure applies

The population forms the basis for the figure. The inclusion and exclusion criteria have an impact to whom or to what the figure applies. The interpretation of the figure needs to be supported by the knowledge of the exact population the figure applies to.

#### The geographical area to which the figure applies

The geographical area concerns the location to which the figure is generalisable. While the geographical area has a large impact on the figure, this characteristic is easily miscommunicated. A figure which is valid for one geographical area may not be applied to another.

#### The time period to which the figure applies

The time period to which the figure applies relates to the question on whether a figure is relevant to the time in which it is reported. Often, a time period to which the figure applies is not mentioned in a secondary publication, implicitly assuming that the figure applies to the present. Whether a figure that is, for example, 2 years old is still relevant at the present time differs strongly between subjects and the aim for which it is used. Furthermore, experience reveals that figures which are counted at one point in time are often confused with longitudinal figures, i.e. point-prevalence and year-prevalence.

#### The process of counting and measuring

The process of counting and measuring is a broad theme covering different methodologies used to construct a figure, and the practical considerations of these methodologies. For each methodology, the main strengths and weaknesses need to be understood in order to interpret the figure.

### Repetition of data collection

Furthermore, the repetition of data collection is considered in this theme. A figure derived from repeated or continuous data collection may be updated over time, or can be compared with earlier figures derived from the same data collection source.

### Sampling

Often, a sample of the population is used. A sample should be large and varied enough to represent the entire population. Statistical reliability increases with sample size and if the sample is relatively small, the reader should be careful with attaching value to it.

### Registries

Registries are used as the basis for many figures on health and healthcare. Here, the way the data is registered, and who registers the data, is of importance. The quality of a registry, and thus the figure derived from it, depends on the completeness of data and the care with which the data is entered.

### Surveys

Questions asked and answers given to a survey determine the eventual figure. Furthermore, these questions should be carefully deduced to conclusions on the subject.

### Direct observations

Data can be obtained through measurements made by researchers or field workers, or in other words, through direct observations. In some cases, this method has an advantage over a survey, but this may depend on the measurement instrument and the care with which the researcher measures. Obviously, many factors, for instance, patient experiences, cannot be easily observed.

### Modelling

If a figure cannot be constructed through empirical methods, the possibility of modelling exists. In modelling, many figures are used for which assumptions are made by the modeller. These assumptions should be well grounded and transparent in order to interpret the reliability of the figure. The plausibility of certain assumptions may often be difficult to judge for many readers.

### The FIAT-Health

The FIAT-Health was constructed out of the eight themes relevant to the interpretation of figures on health and healthcare. The inclusion of all features was decided upon during an iterative process through which the research group thoroughly examined each item included in the tool. Five draft versions of the tool were reviewed by the sounding board. The sounding board gathered five times in order to review the emerging tool, and four draft versions were presented and fine-tuned based on the received feedback. Consensus was reached on the fifth version. In the FIAT-Health, a distinction is made between the primary publication, which is the source in which the figure is reported for the first time, and the publication in which the figure is assessed, which can be both a publication referring to a figure from a primary publication or the primary publication itself.

The characteristics relevant to the interpretation of figures on health and healthcare are addressed through a closed question format with directed routing. As multiple definitions can be applied to the terms used in the FIAT-Health, all terms and phrasing of the questions were discussed extensively and approved by both the research group and the sounding board.

The FIAT-Health consists of 13 main questions with sub-questions, numbered 1–13, and two statements, numbered 14 and 15. Questions 1–13 guide the interpretation and assessment of the figure, and consist of two types of questions, namely characterisation questions and assessment questions; the former are neutral, whereas the latter express a value judgment. The characterisation questions will guide the user into understanding the figure without directing towards an assessment, while assessment questions do provide a distinction between a positive and negative answer. Three types of assessment questions are used, (1) factual questions, which relate to the accessibility of information on a particular theme; (2) assessment questions, for which the user is asked to give a value judgment on a particular theme; and (3) correspondence questions, which relate to the correspondence between the primary publication and the publication in which the user is assessing the figure, which is not relevant if the figure is assessed in a primary publication.

Completing all questions results in a structured, 1-page overview of answers given to questions 1–13. This structured overview supports users to order their thoughts about the strengths and weaknesses of the figure. Through statements 14 and 15, users respectively assess the correctness of the figure and assess the appropriateness of the report of the figure.

### Answer format

Characterisation questions are answered using a dichotomous yes/no scale. These answers do not give a positive or negative value to the answer. Factual and correspondence questions are also answered using a dichotomous yes/no scale, on which the answer ‘yes’ is continuously positive and ‘no’ is negative. Answers to assessment questions are given through a numerical 4-point Likert scale (1 = negative, 4 = positive). To support the user of the FIAT-Health in assessing the figure, a 4-point scale was chosen, avoiding a middle option to stimulate users in forming an assessment [56]. Concluding statements 14 and 15 are accompanied by a 5-star scale, which has been found to be easy to understand in previous studies [57]. Furthermore, to guide the user of the FIAT-Health, the questionnaire is accompanied by a user guide including explanations of all questions.

The glossary used in the FIAT-Health is described in Table 1. The FIAT-Health 1.0 questionnaire, as provided in Table 2, was developed in Dutch. The translation to English was conducted through a forward-back translation by two bilingual translators. Discrepancies in translation were discussed within the research group and the translators until agreement was reached [55].

**Table 1:** Glossary of the FIAT-Health

Glossary	
Figure	The reported result in numbers of the process of counting and measuring
Primary publication	The medium in which the figure was first made public, for example, a report, database, website or scientific publication
Author of the primary publication	The person/persons or organisation who described the figure in the primary publication
User	The person or organisation who wants to understand, cite or distribute the figure
Subject	The aspect of health or healthcare to which the figure refers, for example, a disease, lifestyle factor or treatment
Unit	The measure in which the figure is expressed, for example, persons, Euros, days or kilometres
Population	A collection of units, which can consist of people or objects
Process of counting	The manner in which a quantity is determined, for example, through a registration or direct observations
Process of measuring	The manner in which the presence or size of the subject is determined through predefined values, for example, the measurement of weight based on a scale or based on a survey question

**Table 2:** The FIAT-Health 1.0 questionnaire

Questionnaire FIAT-Health 1.0			
	Question	Answer	Routing
	What figure would you like to assess? (Provide the phrase in which the figure is mentioned)	[−−]	
	Question 1. Origin of the figure		Answer question 1a
1a.	Is the publication in which the figure is reported a primary publication?	Yes/no	If yes, go to question 2; if no, answer question 1b and 1c
1b.	Is the primary publication known?	Yes/no	
1c.	Is the primary publication verifiable?	Yes/no	
	Question 2. Credibility of the figure		Answer question 2a and 2b
2a.	How do you rate the credibility of the primary publication?	Scale	
2b.	How do you rate the independence of the author of the primary publication in relation to this particular figure?	Scale	
	Question 3. Expression of the figure		Answer question 3a
3a.	Is the figure expressed in absolute terms?	Yes/no	If yes, answer question 3c; if no, answer question 3b; if the figure is not taken from a primary publication, answer question 3c
3b.	Is the figure expressed in relative terms?	Yes/no	
3c.	Does the figure you are assessing match the figure in the primary publication?	Yes/no	
	Question 4. Subject to which the figure applies		Answer question 4a; if the figure is not taken from a primary publication, answer question 4b
4a.	How do you rate the clarity with which the subject is described in the primary publication?	Scale	
4b.	Does the definition of the subject of the figure you are assessing match the definition of the subject in the primary publication?	Yes/no	
	Question 5. Population to which the figure applies		Answer question 5a; if the figure is not taken from a primary publication, answer question 5b
5a.	How do you rate the clarity with which the population is described in the primary publication?	Scale	
5b.	Does the definition of the population of the figure you are assessing match the definition in the primary publication?	Yes/no	
	Question 6. Geographical area to which the figure applies		Answer question 6a; if the figure is not taken from a primary publication, answer question 6b
6a.	How do you rate the clarity with which the geographical area is described in the primary publication?	Scale	
6b.	Does the geographical area of the figure you are assessing match the geographical area in the primary publication?	Yes/no	
	Question 7. Time period to which the figure applies		Answer question 7a; if the figure is not taken from a primary publication, answer question 7b
7a.	Is the time period in which the units are counted described in the primary publication?	Yes/no	
7b.	Does the time period to which the figure applies match the time period in the primary publication?	Yes/no	
	Question 8 till 13: Methods of counting and measuring		
	Question 8. Data collection		Answer question 8a; if no, answer question 8b. If yes, go to question 9
8a.	Are the data on which the figure is based collected periodically?	Yes/no	
8b.	Are the data on which the figure is based collected only once?	Yes/no	
	Question 9. Sample		Answer question 9a
9a.	Is the figure based on a sample?	Yes/no	If yes, answer question 9b, 9c, 9d and 9e, and then question 10; if no, go to question 10
9b.	Is the sample size known?	Yes/no	
9c.	Is the response known?	Yes/no	
9d.	Were important groups disregarded in the calculation of the figure?	Yes/no	
9e.	How do you rate the representativeness of the sample?	Scale	
	Question 10. Registration		Answer question 10a
10a.	Were the data collected through an existing registration?	Yes/no	If yes, answer question 10b; if no, go to question 11
10b.	Is it known which registration was used?	Yes/no	If yes, answer question 10c; if no, go to question 11
10c.	How do you rate the usability of this registration for the calculation of this specific figure?	Scale	
	Question 11. Survey research		Answer question 11a
11a.	Were the data collected through survey research?	Yes/no	If yes, answer question 11b and 11c; if no, go to question 12
11b.	Are the questions on which the figure is based described precisely?	Yes/no	
11c.	Are the answer categories of the questions described?	Yes/no	If yes, answer question 11d; if no, go to question 12
11d.	How do you rate the conclusion that was made based on the questions and the answer categories?	Scale	
	Question 12. Direct observations		Answer question 12a
12a.	Are the data collected through direct observations?	Yes/no	If yes, answer question 12b; if no, go to question 13
12b.	Is it known how the direct observations took place?	Yes/no	If yes, answer question 12c; if no, go to question 13
12c.	How do you rate the accuracy of the direct observations?	Scale	
	Question 13. Modelling		Answer question 13a
13a.	Was the figure constructed through modelling?	Yes/no	If yes, answer question 13b; if no, go to the final assessment
13b.	Are the assumptions which were made in the model known?	Yes/no	If yes, answer question 13c; if no, go to the final assessment
13c.	How do you rate the plausibility of the assumptions made in the model?	Scale	
	Final assessment Your final assessment of the figure in the primary publication		
14.	The original figure is correct		Rate by giving 1 to 5 stars
	Your final assessment of the publication in which the figure is reported:		
15.	The use of the figure in the report is appropriate		Rate by giving 1 to 5 stars

## Discussion

The purpose of this study was to develop a tool enabling a systematic assessment of publically reported figures on health and healthcare to improve assessment of these figures by policy-makers, managers, journalists, researchers, patients and the general public. The FIAT-Health can be used to assess publically reported figures on health and healthcare, and is recommended to be applied by (1) policy-makers to support their interpretation of figures to guide their decision-making process; (2) knowledge institutes and policy advisers to provide grounded advise on the use of figures on health and healthcare to policy-makers; (3) journalists, bloggers and information officers during the writing of a public report, summary or commentary; and (4) researchers for the translation of their own research to the public.

The tool should enable its user to (1) detect possible causes of bias associated with the construction of a figure; (2) distinguish the weaknesses and strengths associated to the methodology of a figure; (3) recognise in which context a figure may be used; and (4) detect inconsistencies in the communication of the figure reported to the public and in the primary publication. The FIAT-Health is designed to support its user to systematically interpret a figure on health or healthcare, facilitating the assessment of the quality of the figure, and the assessment of the appropriateness of the public report of this figure. The FIAT-Health is a practical tool avoiding extreme details while assuring coverage of all relevant aspects of the construction of the figure. Moreover, the tool should not generate a definite conclusion on the value of a figure, but should serve as a systematic guide for the thinking process of its user.

The importance of the characteristics found in this study is widely recognised to be influencing interpretation of figures, and are key to the reporting of figures on health and healthcare. Particular to this tool is the attention paid towards the clarity of definitions [58–61] and the credibility [21, 29] of the primary publication. Novel to this tool are the questions aimed to characterise the figure, which is often not addressed in any reporting guideline, but has a large influence on how the figure is interpreted [62, 63]. Furthermore, other themes are in accordance with the content of other tools supporting the reporting of quantitative evidence, namely the GATHER statement [39], the Drummond checklist [64] and the STROBE checklist [65]. Like the GATHER statement, the FIAT-Health is meant to improve reporting as well as to serve the information needs of decision-makers. The FIAT-Health is applicable to figures of varying origin. While knowledge usually relates to scientific evidence [66], the FIAT-Health is not limited to scientific evidence, and is thus usable in the many cases where figures are constructed through other means. Therefore, this tool does not dismiss the value of figures found through alternate methods, but allows the balanced interpretation of these figures.

### Strengths and limitations

The main strength of the FIAT-Health is the development through collaboration with its potential users, ensuring the relevance of the content of the tool and its suitability for application in professional settings. As the tool is primarily based on the perspectives of both the research and funding institution, it is likely that the language used as well as its characteristics may be biased towards their research paradigm. The involvement of other experts with a different affinity with the problem might have resulted in fewer items. In subsequent research, a larger number of institutes should be involved to broaden the perspective of the tool. Experts involved in the consultation rounds were sensitive to the problem of inadequate reporting. To avoid bias in the selection of experts, different groups of experts were involved, including junior and senior researchers and knowledge integration specialists. Additionally, journalists were consulted. Researcher bias in the identification of items was avoided through regular interaction with the research group and the sounding board. Furthermore, focus groups may incite ‘group think’, where participants may adapt their opinion to fit the group. This bias was avoided by organising different forms of consultation, including individual interviews during the developmental process.

The development of the FIAT-Health was supported by a sounding board, a method comparable with a nominal group technique involving the review of material received in advance and discussion amongst experts forming consensus [67]. This study deviates from a nominal technique as the sounding board in this study involved several rounds and focussed on a qualitative assessment. Although the FIAT-Health is based upon the assumption that better information on evidence leads towards better decisions, it is not a decision support tool such as the SUPPORT tools [68], which have the goal to guide policy-makers directly.

Fully understanding all methodological characteristics of figures, and overseeing its consequences, is often extremely challenging and time consuming, even for those who work in research. Further, figures communicated in the media are often biased and misunderstood. The FIAT-Health is by no means a panacea that will fully solve this problem. While developing the tool, one of the main challenges was to find the right balance between thoroughness, on the one hand, and practicality and compactness on the other. It may be unavoidable that the tool is too complex and time consuming for some, and too superficial for others. Consequently, the expectation that an average newspaper-reader will invest time in assessing figures using the FIAT-Health is unrealistic; neither will an experienced statistician obtain great revelations using the FIAT-Health; however, the purpose of this research is not to aid these groups of people. The FIAT-Health fills the critical gap between the expert and the news-consumer, consisting of policy-makers and advisors, journalists, managers, patients and those of the general public whose decisions depend on the correctness of a figure and its reporting.

### Steps ahead

Although face and content validity are established, the FIAT-Health 1.0 is not yet tested for user experience and construct validity. Therefore, a validation study will be performed during the next stage of this study. The current paper format of the tool does not facilitate easy usage, creating a possible barrier to the uptake of the instrument. To encourage uptake of the FIAT-Health into practice, an online version is under construction, facilitating the easy and efficient routing of a user through the questions, after which an overview of the answers can be provided. Nevertheless, despite its current practical limitations, the questions provided in the FIAT-Health are highly relevant, and can thus be applied in every-day practice immediately. As such, a sample of the content of the RIVM website ‘de Staat van Volksgezondheid en Zorg’ was systematically assessed using the FIAT-Health 1.0. The implementation of the FIAT-Health in the improvement process of the website is currently being explored.

## Conclusion

The FIAT-Health is a tool enabling systematic assessment of publically reported figures on health and healthcare, to support a better understanding and interpretation of these figures by policy-makers, managers, researchers, patients, and the general public. The use of the tool results in a 1-page overview, representing the main strengths and weaknesses of a figure, filling the gap of scientifically developed methods which support the public reporting of figures on health and healthcare. As few systematic methods are available through which figures on health and healthcare can be interpreted, the FIAT-Health adds a practical approach through which users are better informed and supported in their decision-making processes.

## Abbreviations

FIAT-Health: Figure Interpretation Assessment Tool–Health
RIVM: Dutch National Institute for Public Health and the Environment
